# Supplementary material for: Acceptance and compliance with micronutrient powder (MNP) among children aged 6–23 months in northern Nigeria
Source: PLOS Glob Public Health. 2022 Oct 17;2(10):e0000961. doi: 10.1371/journal.pgph.0000961 (PMC10022258; doi:10.1371/journal.pgph.0000961)
Supplement: S1 Dataset — (ZIP) [file pgph.0000961.s008.zip › S8_Dataset/Spot Checks.zip!/Spot Checks/06072015_A_GA_AD_2_SC a.pdf]

SPOT CHECK RECORDING FORM

State: Adamawa **LGA: ____Ganye___ Community: _Dadiri_**

Name of caregiver (female): Amina Shuaibu

Name or caregiver (male): ………………………

Sex of index child: _Male___ Age of index child (in months): _1 8 months_______

Spot Check Information

1. **Date of MNP distribution to household** (circle option below based on LGA name above)**:**
   - **Kebbi:** BirninKebbi&Jega LGA: **June 16**
   - **Kebbi:** Danko-wasagu: **June 17**
   - **Adamawa:**Song, Ganye: **(June 18)**
   - **Adamawa:**Guyuk **June 19**
2. **Date of Spot Check Observation: ____6/7/2015_____**
3. **Number of sachets remaining**(Ask the caregiver to show you and count the specific number of sachets remaining)**:**

41

4. **Number of sachets that should remainusing one MNP sachet per day** (Analyst only)**:**

43

5. **Number of sachets remaining / number of sachets should be remaining**(Analyst only)**:**

   _____41____ / ___43______ = __95.3__ %

Probes

Question 1. You have **XX** MNP sachets remaining. Explain how easy or difficult it was to use the MNP product on a daily basis for your child 6 – 23 months? (Circle one answer)

Very Difficult **Difficult** **(Easy)** **Very Easy**

Question 2. Describe any specific challenges that you faced that made the MNP difficult to use on a daily basis?
The sachet is hard to unseal. Sometimes I have to use knife or my teeth to tear off the sachet.

Question 3. Describe specific aspects of the MNP product that you or your child enjoyed that made it easier to use on a daily basis?

- His appetite has increased.
- He used to be ill most of time but since I started giving him this MNP he is healthier.
- He had some rashes on his body before I started giving him MNP but it dried up in less than a week of giving him MNP
- He breastfeeds less than before taking the MNP because now he eats more

Question 4. What questions do you have about the MNP based on your experience using it?

- What will happen if I miss a day of administering the MNP?
- When are we getting more MNP?

Question 5. What specific recommendations do you have that we can do as a program to make the MNP easier to use on a daily basis?

We need more of the MNP because we have seen the benefit and when it is brought again there should be adequate enlightenment.
